# Validity of the Aktibipo Self-rating Questionnaire for the Digital Self-assessment of Mood and Relapse Detection in Patients With Bipolar Disorder: Instrument Validation Study

**DOI:** 10.2196/26348

**Published:** 2021-08-09

**Authors:** Jiří Anýž, Eduard Bakštein, Andrea Dally, Marián Kolenič, Jaroslav Hlinka, Tereza Hartmannová, Kateřina Urbanová, Christoph U Correll, Daniel Novák, Filip Španiel

**Affiliations:** 1 National Insitute of Mental Health Klecany Czech Republic; 2 Mindpax s.r.o Prague Czech Republic; 3 Department of Psychiatry The Zucker Hillside Hospital Glen Oaks, NY United States; 4 Department of Psychiatry and Molecular Medicine Donald and Barbara Zucker School of Medicine at Hofstra/Northwell Hempstead, NY United States; 5 Department of Child and Adolescent Psychiatry Charité Universitätsmedizin Berlin Berlin Germany; 6 Department of Cybernetics Faculty of Electrical Engineering Czech Technical University in Prague Prague Czech Republic

**Keywords:** bipolar disorder, symptom monitoring, ecological mood assessment, relapse detection, mobile application, mobile phone

## Abstract

**Background:**

Self-reported mood is a valuable clinical data source regarding disease state and course in patients with mood disorders. However, validated, quick, and scalable digital self-report measures that can also detect relapse are still not available for clinical care.

**Objective:**

In this study, we aim to validate the newly developed ASERT (Aktibipo Self-rating) questionnaire—a 10-item, mobile app–based, self-report mood questionnaire consisting of 4 depression, 4 mania, and 2 nonspecific symptom items, each with 5 possible answers. The validation data set is a subset of the ongoing observational longitudinal AKTIBIPO400 study for the long-term monitoring of mood and activity (via actigraphy) in patients with bipolar disorder (BD). Patients with confirmed BD are included and monitored with weekly ASERT questionnaires and monthly clinical scales (Montgomery-Åsberg Depression Rating Scale [MADRS] and Young Mania Rating Scale [YMRS]).

**Methods:**

The content validity of the ASERT questionnaire was assessed using principal component analysis, and the Cronbach *α* was used to assess the internal consistency of each factor. The convergent validity of the depressive or manic items of the ASERT questionnaire with the MADRS and YMRS, respectively, was assessed using a linear mixed-effects model and linear correlation analyses. In addition, we investigated the capability of the ASERT questionnaire to distinguish relapse (YMRS≥15 and MADRS≥15) from a nonrelapse (interepisode) state (YMRS<15 and MADRS<15) using a logistic mixed-effects model.

**Results:**

A total of 99 patients with BD were included in this study (follow-up: mean 754 days, SD 266) and completed an average of 78.1% (SD 18.3%) of the requested ASERT assessments (completion time for the 10 ASERT questions: median 24.0 seconds) across all patients in this study. The ASERT depression items were highly associated with MADRS total scores (*P*<.001; bootstrap). Similarly, ASERT mania items were highly associated with YMRS total scores (*P*<.001; bootstrap). Furthermore, the logistic mixed-effects regression model for scale-based relapse detection showed high detection accuracy in a repeated holdout validation for both depression (accuracy=85%; sensitivity=69.9%; specificity=88.4%; area under the receiver operating characteristic curve=0.880) and mania (accuracy=87.5%; sensitivity=64.9%; specificity=89.9%; area under the receiver operating characteristic curve=0.844).

**Conclusions:**

The ASERT questionnaire is a quick and acceptable mood monitoring tool that is administered via a smartphone app. The questionnaire has a good capability to detect the worsening of clinical symptoms in a long-term monitoring scenario.

## Introduction

### Background

Bipolar disorder (BD) is a severe mental illness characterized by recurrent depressive, manic, and mixed episodes [1]. The World Health Organization has identified BD as one of the most frequent causes of disability in youth [2]. The existing literature suggests that measurement-based care may be an effective clinical strategy in most psychiatric disorders [3-6], and monitoring of the clinical course of BD is therefore of high clinical and research interest. Several organizations, such as the International Society for Bipolar Disorders, have recommended the implementation of measurement-based care in the treatment of BD [7].

Although behavioral analysis, including actigraphy and smartphone monitoring, is currently a topic of ongoing research [8-11], mood assessment is still the primary source of clinical data for BD status. In clinical practice, both clinician-observed instruments and patient-reported instruments are used to assess the course of BD [9,10,12]. In this paper, we focus on collecting self-reported mood data using a mobile phone app, which provides an opportunity to monitor the mental health status of the patient frequently and at low cost.

### Self-reported Measurement Tools

Self-report instruments have been used by clinicians for decades, and there has been increasing interest in mental health technologies for self-report instruments in the past few years. The World Health Organization has reported that mobile technologies have the potential to transform health care in various medical specialties worldwide [13]. Currently, approximately 76% of adults in advanced economies and about 45% of adults in emerging economies own a smartphone [14], and it is assumed that this proportion will continue to increase in the next few years.

The growing interest in digital monitoring platforms for disease monitoring in BD has resulted in an increased demand for quick and focused symptom self-monitoring tools [15]. Self-monitoring tools have been well received by patients [16], and the general validity of smartphone-based monitoring has been demonstrated [17]. Reports from psychiatric clinics also demonstrate that patients using self-report instruments have a better outcome than patients undergoing usual care without the availability of self-report instruments [12].

As mentioned above, patient-reported assessments are used in clinical practice and research. Patient-reported measures are tools used to assess the patient’s condition based on their subjective perspective. The main benefit is self-administration, which does not require the presence of clinical staff and can take place in the patient’s natural environment. This process increases ecological validity and reduces the cost of data collection. However, these features also increase the requirements for patient adherence, which can, to some extent, be mitigated by smartphone-aided data collection by using notifications, reminders, or interactions with the study staff when there are missing questionnaires.

In BD, as with observer-reported assessments, there are patient-reported measurement tools available that either assess depressive symptoms and manic symptoms together, or only assess one of the mood polarities. In a meta-analysis [12], the authors summarized existing patient-reported measurement tools with high clinical utility scores and reported three tools that assessed only depressive symptoms, four that assessed only manic symptoms, and five that were used to assess both manic and depressive symptoms.

Currently, the most used self-reported measurement instruments that assess both manic and depressive symptoms are the Internal State Scale [18], Multidimensional Assessment of Thymic States [19], Affective Self-Rating Scale [20], National Institute of Mental Health’s (NIMH’s) Prospective Live Chart Methodology-Self [21], ChronoRecord [22], MoodZoom [23], and openSIMPLE [24,25].

For the purposes of long-term monitoring of patients with BD via a mobile app, we found no existing questionnaires that would meet our requirements, that is, (1) a questionnaire with short completion time with no more than 10 questions and only a limited number of options; (2) a questionnaire for assessing both manic and depressive symptoms on comparable scales; (3) a questionnaire designed for weekly sampling; (4) a questionnaire with high sensitivity to subclinical mood changes during remission periods, allowing early detection of deterioration of the clinical state; and (5) a questionnaire that was suitable and validated for user-friendly smartphone delivery.

### This Study and the Aktibipo Self-rating Questionnaire

In this study, we present a novel tool for unassisted, app-based, self-evaluation of mood, the ASERT (Aktibipo Self-rating) questionnaire [26], aimed at the depressive and manic symptoms of patients with BD, following the five principles mentioned earlier.

We aimed to answer the following three principal questions: (1) what is the validity of the questionnaire for measuring depression and mania with respect to the respective standard clinical scales; (2) what is the internal structure of the questionnaire, and what is its consistency; and (3) can ASERT be used for detecting a symptomatic episode (a relapse), as defined by the corresponding clinical scales?

## Methods

### The ASERT Questionnaire

ASERT is a novel questionnaire for the ecological momentary assessment of mood in BD [26]. The questionnaire contains 10 items that map depressive symptoms (4 items), manic symptoms (4 items), and nonspecific symptoms (two items), with 5 possible response levels for each symptom. The observation period is the past week. The questionnaire is available in Czech, English, and German. The English and German versions were validated using back translation. The questionnaire was administered on a weekly basis through a smartphone app developed by Mindpax [27]. The questions and reply options are presented in Table 1. The presented results used the Czech version in a clinical study.

**Table 1:** The three available language versions of the ASERT (Aktibipo Self-rating) questionnaire.

Group and number^a^		English version^b^	German version^c^	Czech version^d^
**Depressive**				
	1	I feel sad, downhearted	Ich fühle mich traurig und niedergeschlagen	Cítím se smutně, sklesle
	2	I do not enjoy anything, and nothing pleases me	Ich genieße nichts und ich habe an nichts Gefallen	Nic mě nebaví, netěší
	3	I have no energy	Ich habe keine Energie	Nemám energii
	4	I feel gloomy and pessimistic about the future	Ich bin bedrückt und pessimistisch über die Zukunft	Budoucnost vidím černě, pesimisticky
**Manic**				
	5	I feel unusually great, optimistic	Ich fühle mich ungewohnt großartig und bin ungewöhnlich optimistisch	Cítím se neobvykle skvěle, optimisticky
	6	I have excess energy	Ich habe einen Überschuss an Energie	Mám nadmíru energie
	7	My thinking is very fast, others cannot keep up with me	Mein Denken ist sehr schnell, andere können mit mir nicht mithalten	Myslí mi to hodně rychle, ostatní mě nestíhají
	8	I need to sleep less than usual	Ich brauche weniger Schlaf als sonst	Potřebuji spát méně, než obvykle
**Nonspecific**				
	9	I feel restless, tense	Ich fühle mich ruhelos und angespannt	Cítím neklid, napětí
	10	I cannot focus	Ich kann mich nicht konzentrieren	Nemohu se soustředit

^a^Usage of the ASERT (Aktibipo Self-rating) questionnaire is subject to a written agreement with Mindpax s.r.o.

^b^Reply options: 0=I do not agree; 1=more likely I do not agree; 2=I probably agree; 3=I agree; 4=I completely agree.

^c^Möglichkeiten: 0=Trifft nicht zu; 1=Ich stimme eher nicht zu; 2=Teils/Teils; 3=Trifft eher zu; 4=Trifft zu.

^d^Možné odpovědi: 0=nesouhlasím; 1=spíše nesouhlasím; 2=asi souhlasím; 3=souhlasím; 4=naprosto souhlasím.

### The AKTIBIPO400 Study

The questionnaire, as well as its validation, is part of the ongoing noninterventional and nonpharmacological AKTIBIPO400 study. The aim of the clinical study is to monitor the physical activity, mood, and clinical state of patients with BD and those of healthy controls using an actigraphy wristband with telemetric data transmission. The participation timeframe was 18 months for patients with BD and 3 months for healthy controls. The study population included men and women between 18 and 60 years of age undergoing standard clinical treatment for BD (International Classification of Diseases-10 diagnosis F31) and remitted at enrollment, meeting the thresholds of the Montgomery-Åsberg Depression Rating Scale (MADRS) [28] sum score ≤9 and the Young Mania Rating Scale (YMRS) [29] sum score ≤12. All study participants in all groups wore a wrist-worn actigraphy device at all times and used the accompanying mobile app (a proprietary system developed by Mindpax) using which they submitted weekly ASERT mood self-reports. Further study details can be found in the study website [30].

The study was approved by the institutional ethical committee of the NIMH (Czech Republic; case number: 101/17) and was conducted in compliance with the Declaration of Helsinki.

### The Mindpax Mobile Health System

**Figure 1:**
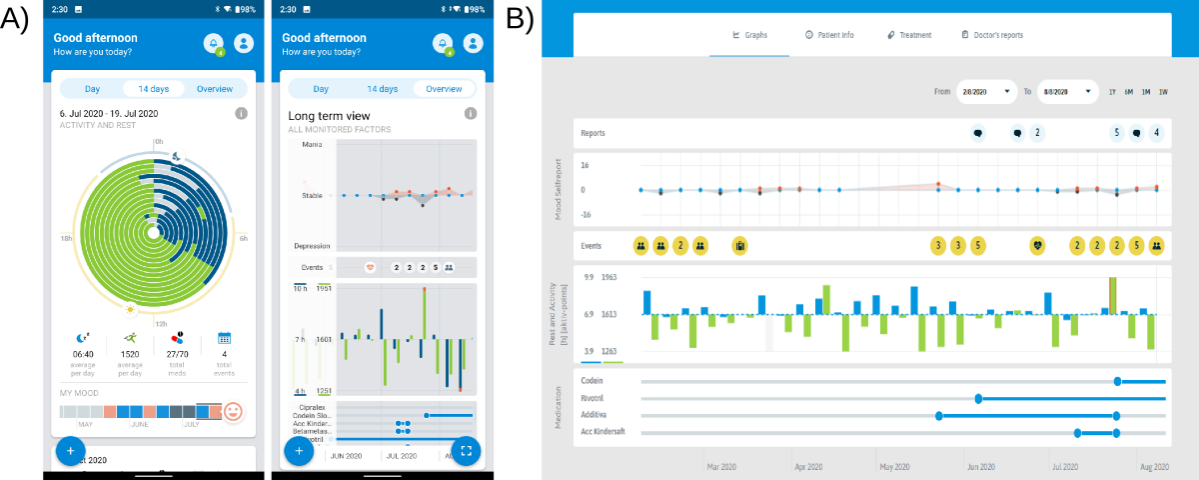
Snapshots from the Mindpax mHealth system. (A) Patient view of the mobile app showing activity and sleep in a spiral graph as well as the time course of mood. (B) Clinician view of the system showing various parameters reported by the patient and measured by wristband actigraphy. mHealth: mobile health.

The study used a mobile health system developed by the Mindpax company, which included a mobile app installed in the participant’s smartphone (Android or iOS) and the Mindpax wristband (see Figure 1 for system overview). The wristband version used in the study included a nonremovable battery with a battery life of approximately 12 months. Epoch-level actigraphy data were aggregated and stored by the wristband and regularly (several times a day) transferred via Bluetooth to the mobile app and through secured connection via mobile data or Wi-Fi to the Mindpax servers for evaluation. To avoid bias or confusion, the participants were blinded to their activity and mood history, in addition to visualizations of sleep length and total daily activity. Figure 1 shows a mobile app version dedicated to end users with full feedback. To ensure the privacy of patient data, the system uses secure data connections (secure shell protocol), and the system accounts are anonymized, with citizen names and clinical details available to the clinical study management team and participating psychiatrists only. The Mindpax system is a class I medical device notified by the Czech State Institute for Drug Control under reference number 00904675, and the company undergoes a regular certification process under International Organization for Standardization (ISO) 13485.

### Clinical State Assessment

For the purposes of this study, only the participants in the core group of the AKTIBIPO400 study [30] were included: those with a diagnosis of BD and bipolarity index >50 [31,32], as evaluated by an institutional psychiatrist during the initial visit to the NIMH at enrollment. The patients were assessed monthly using the MADRS and YMRS clinical scales via telephone interviews by trained psychologists. Additional information about clinical episodes, hospitalizations, and work status was collected retrospectively every 6 months from the caregiving psychiatrists.

Clinical scales were used as the primary measure of the clinical state. For relapse detection, we used the definition of a depressive relapse as the sum of MADRS≥15, and for a manic relapse as the sum of YMRS≥15; the nonrelapse state (remission) was defined as the sum of MADRS<15 or the sum of YMRS<15, in accordance with the International Society for Bipolar Disorders task force definition of an episode more severe than subsyndromal depression or mania [7].

### Data Preprocessing

The different intervals of the sampling period for the self-reported ASERT questionnaire (weekly) and the clinical scales (monthly) pose a challenge to evaluating the validity of the ASERT questionnaire. Therefore, we created a matched data set, including only ASERT responses no further than 3 days from the nearest MADRS and YMRS assessment event of a given participant, thus containing all ASERT responses in a week-long window around each phone assessment. MADRS and YMRS assessments without such defined matching ASERT responses were excluded from the data set. All matchings were unique, and neither of the two clinical scale assessments was matched to more than one self-report and vice versa. No missing values in individual question responses were possible, as the mobile app allowed only complete questionnaires to be submitted. All clinical scale assessments were completed. Therefore, no imputation of missing values was needed, and completely missing scales and questionnaires were not imputed.

### The Internal Structure and Basic Properties of the ASERT Questionnaire

We used the principal component analysis [33] to validate the content, that is, the assumed three-factor structure of 4 depression-related questions, 4 mania-related questions, and 2 nonspecific questions of the ASERT questionnaire. Cronbach *α* [34] was calculated to provide a consistency estimate for each factor.

We estimated the ASERT response rate of each patient to the platform as the number of filled-in self-reports divided by the expected number of filled-in reports (ie, the number of weeks spent in the study).

### A Comparison Between ASERT Responses and Clinical Scales

#### Linear Mixed-Effects Models

The convergent validity between the ASERT questionnaire and the corresponding clinical scales was assessed using linear mixed-effects (LME) models [35], which consisted of fixed effects that quantified the dependency shared across the group, and random effects that quantified individual effects. For these analyses, data from a single patient were considered dependent; therefore, random effects parameters (random intercepts and slopes) were estimated at the individual patient level.

#### Correlation

Apart from the LME, we also calculated Pearson correlation coefficients to measure the association between the ASERT depression and mania item responses and the MADRS and YMRS, respectively.

In addition, we also computed correlations between selected individual items from the clinical scales and matching items in the ASERT questionnaire. As interindividual variation may lead to biased results when estimating correlation coefficients, we used a simple 2-step procedure to estimate the correlation coefficient in cases with substantial individual variation. In the first step, we computed the correlation coefficient for each patient separately. In the second step, we averaged the individual correlation coefficients to estimate the group-level correlation coefficient. Because each patient is represented by a different number of observations, due to the ongoing state of the AKTIBIPO400 study, we decided to introduce weighting into the second step of the procedure by assigning a weight that was proportional to the number of observations available for each patient.

#### Statistical Assessment

To assess the statistical significance of the estimated LME fixed-effects coefficients and group correlation coefficients, we performed a case bootstrap with 10,000 resamples. In each iteration, we sampled the data on both levels of the structure, which means that for each simulation, we took a random sample with replacement on the level of patients, and subsequently we sampled with replacement from the observations in each patient subset.

### Detection of Scale-Based Relapses From the Self-reports

After evaluating the validity of the questionnaire, we assessed the performance of the questionnaire in identifying a relapse based on a score of ≥15 on the MADRS or YMRS scale. The MADRS or YMRS scores of <15 were considered a nonrelapse (interepisode or remission) state. Only patients with records of both relapse and nonrelapse states were included in the subset for this task. Similar to the comparison of the ASERT questionnaire and the clinical scales described above, we used a generalized mixed-effects model. In this task, we used a logistic mixed-effects model due to the binary outcome (relapse or nonrelapse).

Two separate models were trained and evaluated for the detection of depressive and manic relapses. To increase the predictive robustness of the relapse detection model, we extended the model inputs by adding the values of the ASERT questionnaire responses one week before the clinical scale assessment (matched in the window 4-10 days before the phone interview), and the ASERT responses 2 weeks before the clinical scale assessment (matched in the window 11-17 days before the phone interview).

As *false alarms* (ie, lack of specificity) significantly affect the usability of a relapse detection model, we used the receiver operating characteristic on the training set to modify the detection threshold of the trained model to achieve the desired specificity of 90%.

### Model Validation and Model Selection

To obtain an unbiased estimate of the model relapse detection performance, we used a standard split-validation design in a ratio of 70:30. In this scenario, the data set is divided randomly into two subsets: the training data set (70%) on which the model is trained, and the testing data set (30%) on which the model is evaluated. The data splitting procedure is performed with respect to individual patients, resulting in each patient being represented by the same ratio of his or her data in the training and testing sets.

The model performance criteria were computed for the testing set. We chose relapse detection accuracy, area under the receiver operating characteristic curve (AUROC), sensitivity, and specificity as a set of model performance measurements. We repeated the split-validation procedure 999 times with randomly chosen splits to obtain a model performance distribution rather than a point estimate.

The best-scoring model was selected using a stepwise comparison procedure. The sequence of model structures consisted of a simple model without random effects (a logistic regression model), followed by models with various random effect structures and various predictor variables (see Table S1 in Multimedia Appendix 1 for a more detailed description of the models). In each comparison, the pair consisting of the basic (simpler) model and the more complex model was repeatedly trained and tested on randomly selected training and testing sets. Performance measures were recorded and compared in a pairwise manner. The basic model was rejected as being inferior to the more complex model if the performance measures were better in a substantial number of data set splits (≥90% of the random simulations).

## Results

### Data Description

The original data set contained 8438 ASERT self-reports from 103 patients and 2186 records of clinical scales (MADRS, YMRS) from 107 patients. For ASERT validation, we used only a subset of the ASERT self-reports for which a concurrent record of the clinical scales was available. As described above, the clinical scale and the self-report were considered concurrent or temporally matching if they were obtained no more than 3 days apart.

After applying these constraints to the data, we obtained 2159 paired records (ie, clinical scales with corresponding ASERT responses) from 99 patients, which were used for the questionnaire validation and for evaluating the internal structure of the questionnaire. The participant characteristics for this data set are summarized in Table 2, which summarizes patients’ general demographics, psychometrics, and medications (for a full list of medications refer to Table S2 in Multimedia Appendix 1).

The median completion time of the ASERT, measured by the application and stored in the database, was 24 seconds. The average response rate was 78.1% (SD 18.3%).

**Table 2:** Demographic and diagnostic characteristics of the validation data set.

Demographics category				All patients (n=99), n (%)		Relapse detection (subset: depression; n=51), n (%)		Relapse detection (subset: mania; n=27), n (%)
**General demographics**								
	Patients^a^, n (%)		99 (100)		51 (52)^b^		27 (27)^b^	
	Age^c^ (years), mean (SD)		37.7 (11)		38.0 (10.6)		35.7 (10.5)	
	**Sex^c^, n (%)**							
		Female	60 (61)		33 (65)		21 (78)	
		Male	39 (40)		18 (35)		6 (22)	
**Psychometrics**								
	Days of follow-up^a^, mean (SD; range)		754.0 (266.4; 64-1116)		800.5 (229.6; 390-1116)		800.4 (236.5; 390-1113)	
	Response rate^a^, mean (SD; range)		0.78 (0.18; 0.16-1)		0.76 (0.18; 0.32-0.98)		0.79 (0.16; 0.47-0.98)	
	Bipolarity index^c^, mean (SD; range)		68.7 (10.6; 50-90)		69.0 (10.8; 52-90)		69.5 (10.4; 52-90)	
	MADRS^d^, mean (SD; range; N)		4.78 (6.6; 0-48; 2159)		7.07 (7.9; 0-48; 1121)		5.9 (8.0; 0-48; 635)	
	YMRS^e^, mean (SD; range; N)		2.14 (4.1; 0-35; 2159)		2.76 (4.7; 0-35; 1121)		4.45 (6.1; 0-35; 635)	
**Medication, n (%)**								
	Antidepressant		43 (43)		30 (59)		13 (48)	
	Antipsychotic		76 (77)		38 (75)		20 (74)	
	Mood stabilizer and anticonvulsant		81 (89)		46 (90)		23 (85)	

^a^The items referring to the end of the study (number of patients, days of follow-up, and response rate).

^b^n=99.

^c^The items obtained at the beginning of the study (age, sex, and bipolarity index). The items without any markup were collected throughout the study.

^d^MADRS: Montgomery-Åsberg Depression Rating Scale.

^e^YMRS: Young Mania Rating Scale.

### Content Validity of the ASERT Questionnaire

The principal component analysis of the ASERT responses showed two dominant components—*pc_1_* with 50.3% explained variability and *pc_2_* with 21% explained variability (Tables 3 and 4). The first principal component relates to the depression-related questions (*pc_1_* high values of loadings for questions 1-4) together with nonspecific questions (*pc_1_* high loadings for questions 9 and 10). The second principal component summarizes the mania-related questions (*pc_2_* high values of the loadings for questions 5-8) and nonspecific questions (*pc_2_* high values of loadings for questions 9 and 10), but their loading coefficients were smaller than those of the main mania-related questions. Each of the remaining principal components explained <7.4% of the variability.

The Cronbach *α* values showed high consistency for the sum of the depression-oriented questions Q1-Q4 (Cronbach *α*=.92; if nonspecific, Q9 and Q10 were also added, the value remained at Cronbach *α*=.92), and for the mania-oriented questions Q5-Q8 (Cronbach *α*=.85; if Q9 and Q10 were added, the consistency dropped to Cronbach *α*=.72). The two nonspecific questions Q9 and Q10 together also showed high consistency (Cronbach *α*=.85).

The results indicated that (1) the ASERT questionnaire demonstrates the intended two-factor structure with highly consistent components and (2) the two nonspecific questions are more correlated with the depressive cluster. Therefore, we concluded that it was reasonable to focus only on the summary characteristics (the sum of the questions about depression, the sum of the questions about mania, and the sum of the nonspecific questions), rather than the individual items, in our further analyses.

**Table 3:** Loadings of the principal component analysis of the ASERT^a^ questionnaire self-reports.

ASERT questionnaire question number	*PC^b^_1_*	*PC_2_*	*PC_3_*	*PC_4_*	*PC_5_*	*PC_6_*	*PC_7_*	*PC_8_*	*PC_9_*	*PC_10_*
Q1	0.433	—^c^	0.282	–0.281	0.111	–0.423	0.229	–0.178	–0.580	–0.190
Q2	0.425	—	0.310	–0.177	—	–0.111	0.147	–0.266	0.752	0.109
Q3	0.427	–0.135	0.207	0.805	0.229	0.136	—	0.108	–0.101	0.113
Q4	0.389	—	0.213	–0.385	–0.221	0.420	–0.435	0.481	—	—
Q5	—	0.506	0.288	0.236	–0.388	–0.101	–0.343	–0.261	—	–0.506
Q6	—	0.493	0.239	—	–0.163	—	—	–0.120	–0.181	0.787
Q7	—	0.415	0.145	—	—	—	0.614	0.608	0.128	–0.201
Q8	—	0.428	—	–0.188	0.805	0.255	–0.179	–0.149	—	–0.107
Q9	0.371	0.261	–0.586	—	—	–0.533	–0.292	0.238	0.124	—
Q10	0.397	0.204	–0.479	—	–0.242	0.502	0.347	–0.352	–0.110	—

^a^ASERT: Aktibipo Self-rating.

^b^*PC*: principal component.

^c^Values with an absolute value lower than 0.1 are not shown.

**Table 4:** Summary statistics of the principal component analysis of the ASERT (Aktibipo Self-rating) questionnaire self-reports.

Summary statistic	*PC^a^_1_*	*PC_2_*	*PC_3_*	*PC_4_*	*PC_5_*	*PC_6_*	*PC_7_*	*PC_8_*	*PC_9_*	*PC_10_*
SD	1.378	0.890	0.527	0.416	0.397	0.341	0.327	0.322	0.271	0.267
Proportion of variance	0.503	0.210	0.074	0.046	0.042	0.031	0.028	0.028	0.020	0.019
Cumulative proportion of variance	0.503	0.714	0.787	0.833	0.875	0.906	0.934	0.962	0.981	1

^a^*PC*: principal component.

### Convergent Validity of the ASERT Depression and Mania Items With the MADRS and YMRS, Respectively

Following the results of the previous section, we modeled the relationships between the clinical scales and ASERT self-reports using two LME models with random slopes and random intercepts for individual patients.

The first model showed a significant linear relationship between the sum of the depression-related and nonspecific questions of the ASERT questionnaire (DEPNSP) and the sum of the MADRS scale, with the slope *β_DEPNSP_*=.87 (*P*<.001) and the intercept *β_0_*=.71 (*P*<.001). The fixed-effects coefficient value means that a unit change in the ASERT depressive and nonspecific subscore corresponds to a 0.87 increase in the MADRS sum score on the study population level. The second model linked mania-related questions to the sum of the YMRS scale and showed a significant association, with a slope of *β_MAN_*=.73 (*P*<.001) and intercept *β_0_*=1.05 (*P*<.001). The fixed-effects coefficient value means that a unit change in the ASERT manic subscore corresponds to a 0.73 increase in the YMRS sum score on the study population level.

Alternatively, using Pearson correlation coefficient, the depression-related subscores of ASERT showed a significant correlation with the sum of MADRS, and the mania-related subscores also showed a significant correlation with the sum of the YMRS scores. Specifically, the weighted group correlation coefficient was *ρ_wgr_*=0.53 (*P*<.001) for the relationship between the MADRS scale and the depression-related and nonspecific components of the ASERT questionnaire and the weighted group correlation coefficient was *ρ_wgr_*=0.32 (*P*<.001) for the YMRS and the mania-related subset of ASERT. For the relationship between the individual ASERT item responses and matching items from the MADRS and YMRS, see Table 5.

**Table 5:** The correlation between Aktibipo Self-rating questionnaire subscores and the total scores of the Young Mania Rating Scale and Montgomery-Åsberg Depression Rating Scale clinical scales and the correlation between individual items of the Aktibipo Self-rating questionnaire and corresponding items from the Montgomery-Åsberg Depression Rating Scale and Young Mania Rating Scale clinical scales.

Subset of variables		Group correlation coefficient-weighted^a^	*P* value
**ASERT^b^ questionnaire depression and mania subscores versus sum of MADRS^c^ or YMRS^d^**			
	MADRS: Sum of questions∼ASERT: sum of questions about depression	0.51	<.001
	MADRS: Sum of questions∼ASERT: sum of questions about depression and nonspecific questions	0.53	<.001
	YMRS: Sum of questions∼ASERT: sum of questions about mania	0.32	<.001
	YMRS: Sum of questions∼ASERT: sum of questions about mania and nonspecific questions	0.25	<.001
**ASERT questionnaire individual items versus MADRS or YMRS selected items**			
	MADRS: 2. sadness (subject)∼ASERT: 1. sadness	0.49	<.001
	MADRS: 9. pessimism∼ASERT: 4. future	0.41	<.001
	YMRS: 2. energy (+)∼ASERT: 6. energy (+)	0.40	<.001
	YMRS: 7. speech and thinking disorders∼ASERT: 7. acceleration	0.46	<.001
	YMRS: 4. sleep∼ASERT: 8. sleep	0.31	<.001
	MADRS: 3. internal tension∼ASERT: 9. unrest	0.31	<.001
	MADRS: 6. disturbance of concentration∼ASERT: 10. concentration	0.46	<.001

^a^Weighted group-level correlation coefficients (ie, taking into consideration the different sample sizes for different patients, the weights are proportional to the number of observations for each patient) for sums of the clinical scales and of the ASERT (Aktibipo Self-rating) questionnaires and for a more detailed view of the relationship between the individual items of the clinical scales and their counterparts in the ASERT questionnaire.

^b^ASERT: Aktibipo Self-rating.

^c^MADRS: Montgomery-Åsberg Depression Rating Scale.

^d^YMRS: Young Mania Rating Scale.

### Relapse Detection Data

Applying the additional constraints on the matched data for relapse detection resulted in the addition of 2069 paired self-reports 1 week before the clinical scale assessments and of 2046 self-reports 2 weeks before the clinical scale assessments.

For depression, 51 patients had data on both relapse and nonrelapse with 1121 paired observations for the coinciding MADRS scale and self-report, 1054 observations from the previous week, and 1072 observations from 2 weeks before. From the 1121 observations, 195 fulfilled the criteria for relapse (MADRS≥15).

For mania, 27 patients had data on both relapse and nonrelapse with 635 paired observations for the coinciding YMRS scales and self-reports, 591 observations from the previous week, and 604 observations from 2 weeks before. Of the 635 clinical scales, 63 fulfilled the criteria for relapse (YMRS≥15).

### ASERT-Based Relapse Detection

For depression relapse (MADRS≥15), the best-performing model from the model selection included the sum of the depression-related questions, together with the nonspecific questions from the current week and from the previous week in the fixed-effects part, and random intercepts for each patient in the random effect part. This was the last model showing any improvement in the succession of hierarchically nested models in the model validation and model selection section and is listed in Table S1 (Multimedia Appendix 1), achieving an accuracy of 88.3%, an AUROC of 0.932, sensitivity of 79.5%, and specificity of 90.2% on the training data set and an accuracy of 85%, AUROC of 0.880, sensitivity of 69.9%, and specificity of 88.4% on the testing data set (Table 6). Inclusion of the ASERT depression score 2 weeks before the scale did not significantly improve this model.

The best-performing model for mania relapse (YMRS≥15) used the sum of mania-related questions from the current week in the fixed-effects part and random intercepts in the random effect part, achieving an accuracy of 88.6%, AUROC of 0.901, sensitivity of 71.3%, and specificity of 90.6% on the training set and an accuracy of 87.5%, AUROC of 0.844, sensitivity of 64.9%, and specificity of 89.9% on the testing set (Table 7). Inclusion of the ASERT mania subscore from the previous week or 2 weeks before the scale did not significantly improve this model.

**Table 6:** Performance of the ASERT (Aktibipo Self-rating) questionnaire in detecting depressive relapse^a^.

Set	Accuracy	AUROC^b^	Sensitivity	Specificity
Training	0.883	0.932	0.795	0.902
Testing	0.850	0.880	0.699	0.884

^a^For depressive relapse, the results of the best-scoring logistic mixed-effects model in the detection of depression relapse from the sum of the depression-related questions and the nonspecific questions from the current week and from the previous week, as follows: MADRS≥15 ~ ASERT_DEPNSP,current week_ + ASERT_DEPNSP,previous week_ + (1 | ID_patient_). MADRS: Montgomery-Åsberg Depression Rating Scale; DEP: depression; NSP: nonspecific question; (1 | ID_patient_): random intercept. Note that the detection thresholds for all models were adjusted to a specificity of 90% by using the receiver operating characteristic curve of the training set.

^b^AUROC: area under the receiver operating characteristic curve.

**Table 7:** Performance of the ASERT (Aktibipo Self-rating) in detecting manic relapse^a^.

Set	Accuracy	AUROC^b^	Sensitivity	Specificity
Training	0.886	0.901	0.713	0.906
Testing	0.875	0.844	0.649	0.899

^a^For manic relapse, results of the best-scoring logistic mixed-effects model in the detection of mania relapse used the sum of the mania-related questions from the current week, as follows: YMRS≥15 ~ ASERT_MAN,current week_ + (1 | ID_patient_). YMRS: Young Mania Rating Scale; MAN: mania; (1 | ID_patient_): random intercept. Note that the detection thresholds for all models were adjusted to a specificity of 90% by using the receiver operating characteristic curve of the training set.

^b^AUROC: area under the receiver operating characteristic curve.

## Discussion

### Principal Findings

The ASERT questionnaire for ecological momentary mood assessment was designed to provide a quick and scalable way to report the self-perceived mood of patients affected by BD via a smartphone app, including mania-related symptoms and depression-related symptoms. We evaluated the content and convergent validity, internal structure, and consistency of the questionnaire, using an extensive data set consisting of 99 patients with a mean follow-up duration of almost 2 years, with weekly ASERT assessments and monthly YMRS and MADRS phone interviews.

The analysis of the internal structure using principal component analysis confirmed the assumed structure of 4 depression-oriented questions (Q1-Q4), 4 mania-oriented questions (Q5-Q8), and 2 nonspecific questions (Q9 and Q10) with high internal consistency (Cronbach *α*≥.85). Although higher scores on the nonspecific questions (Q9 and Q10) were associated with higher depression scores and higher manic scores, nonspecific questions were more consistently associated with depressive scores.

Although the two nonspecific questions (Q9: “I feel restless, tense” and Q10: “I cannot focus”) did not increase consistency when summed up with the depressive questions, they increased the accuracy of the mixed-effects model in comparison with the MADRS and increased accuracy in the detection of depressive relapses. This improved performance is likely due to the matching items being present in the MADRS scale—the items number 3 (inner tension) and 6 (concentration difficulties)—which have previously been shown to form a separate cognitive-anxiety cluster [36] or were used as a proxy for anxiety [37]. However, both variants, including or excluding nonspecific questions, are reasonable when estimating the depressive state of the patient and may be used depending on the intended range of symptoms studied.

This symptomatic dimension, mirrored by both nonspecific ASERT questions and MADRS items 3 and 6, might be of specific heuristic value in further parsing the neurobiological heterogeneity of mood disorders. In a large sample of subjects with affective disorders, the presence of restlessness and distractibility identified subgroups with an increasing likelihood of BD diagnosis [38]. These findings provide an important empirical confirmation that these symptoms may play a role in discriminating between unipolar disorder and BD. This finding is of particular relevance as patients with BD with comorbid anxiety and rapid mood switches might represent a genetically distinct subtype of BD [39]. Primarily, continuous monitoring of anxiety might be of clinical significance due to the association of comorbid anxiety with greater severity, recurrence, and overall impairment in BD [40,41]. On the basis of these arguments, we decided to keep the nonspecific questions as part of the ASERT.

The main advantages of the ASERT questionnaire are its simple structure and coverage of the main symptoms of BD. The simple structure of ASERT allows it to be administered via a mobile app and to be evaluated quickly (the median filling time was 24.0 seconds), which should contribute positively to adherence to the self-report by the patients; in our case of a long-term follow-up, the mean response rate was 78.1%, which is comparable with previous long-term self-assessment studies [23]. This brevity and efficiency comes at the cost of a decreased level of detail in the description of the mood than can be provided by more extensive questionnaires, such as the Internal State Scale [18] with 17 items, ranging from 0 to 100; the Multidimensional Assessment of Thymic States [19] with 20 items, ranging from 0 to 10; or the Affective Self-Rating Scale [20] with 18 items, ranging from 0 to 4. However, ASERT may arguably provide a higher level of detail than other less granular scales, aimed at both depression symptoms and manic symptoms, such as the NIMH Prospective Live Chart Methodology-Self [21], which has only two items, ranging from –4 to +4, and the ChronoRecord [22], which has six questions, ranging from 0 to 100. However, both of these scales are aimed at a shorter time frame of the past 24 hours, rather than the past week, as in the case of ASERT. This is also the case for the openSIMPLE platform for psychoeducation [24,25], where the patient rates their state in 5 visual analog scales. The daily assessments were compounded using more comprehensive weekly questionnaires.

A comparison between the ASERT subscores and the relevant clinical scales, using an LME model, showed a highly significant association both for the sum of the depression-oriented questions (Q1-Q4) versus the sum of the coincident MADRS scale (*P*<.001) and also for the sum of the mania-oriented questions (Q5-Q8) versus the sum of the coincident YMRS scale (*P*<.001). In addition, many of the ASERT items can be clearly and significantly mapped to the corresponding items from the MADRS or YMRS scales, which increases the interpretability of the collected data.

**Figure 2:**
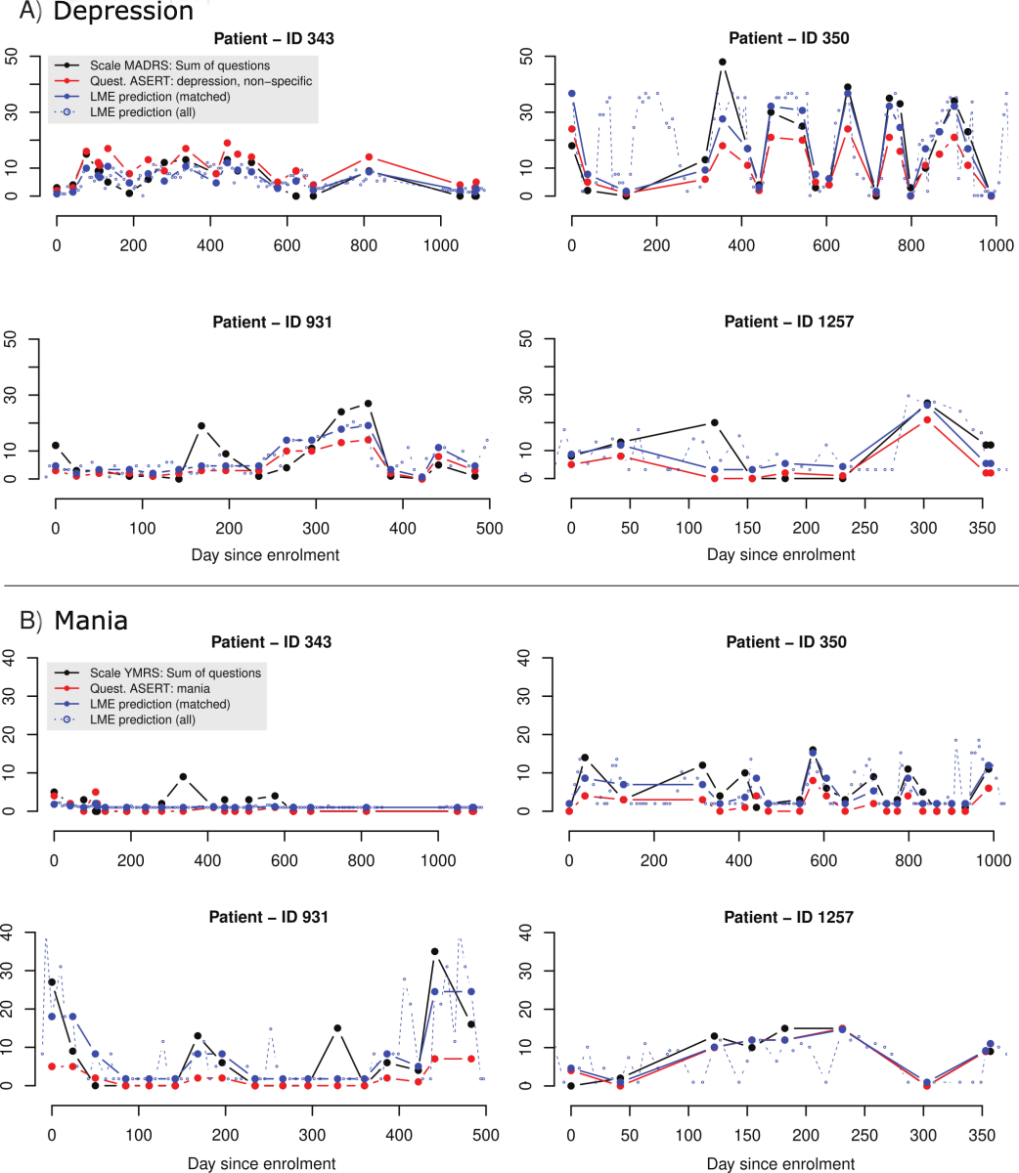
Time courses of clinical scales (MADRS and YMRS) and ASERT questionnaire subscores (depressive and nonspecific, manic) of 4 selected patients, demonstrating the ability of the patients to report their mood. The observations from the presented data set are shown in full lines. The dashed lines show additional ASERT questionnaire responses, unmatched to a near MADRS or YMRS for (A) depression and (B) mania. Full lines: black, MADRS or YMRS; red, ASERT depressive and nonspecific subscore or manic subscore; blue, model estimate of MADRS or YMRS; dashed line, model estimate on nonmatched assert questionnaires. ASERT: Aktibipo Self-rating; LME: linear mixed-effects model; MADRS: Montgomery-Åsberg Depression Rating Scale; YMRS: Young Mania Rating Scale.

Self-reports measure a different perspective on a patient’s current symptoms than that offered by the clinician interview-based scales. Self-reports are subjective and may be biased by impaired insight during symptomatic periods, especially during mania [42]. However, according to a recent review, several existing self-assessment tools for mania showed high clinical validity [43]. In addition, ASERT contains fewer items than MADRS and YMRS. These two reasons are likely responsible for the imperfect, weighted Pearson correlation coefficients, showing a *ρ_wgr_*=0.53 (*P*<.001) for the ASERT depressive and nonspecific subscores compared with the MADRS total score and *ρ_wgr_*=0.32 (*P*<.001) for the manic subscore compared with the YMRS total score. This result indicates that an approximately 30-minute clinical interview cannot be replaced by a 30-second self-report. However, there is a clear difference in the burden on the patient as well as in the collection cost. This difference makes it possible to monitor the course of a patient’s symptoms frequently, without the need for clinician involvement. Figure 2 demonstrates the high overlap between the output of the ASERT-based mixed-effects model and the MADRS or YMRS, as well as its ability to capture additional mood changes between the MADRS and YMRS evaluations.

### ASERT-Based Relapse Detection

In addition to the linear relationship between the ASERT self-report and clinical rating scales, we evaluated the ability of ASERT to identify a relapse defined by MADRS≥15 for depression and YMRS≥15 for mania. The dichotomized MADRS and YMRS scores classified into remission and relapse were modeled using a logistic mixed-effects model. In contrast to the LME models used in the statistical comparison, we used more explanatory variables, including ASERT subscores from the previous weeks. The best-scoring model was selected using a repeated randomized split-validation procedure. The results for the separate models for depression and mania showed high accuracy in relapse detection. For depression, the test set accuracy for a model using the ASERT response from the same and the previous weeks was 85% (sensitivity 69.9%; specificity 88.3%; AUROC 0.880), and for mania, the accuracy of the best model using same-week data was 87.5% (sensitivity 64.9%; specificity 89.9%; AUROC 0.844). The high AUROC values show the good detection capabilities of the model even for highly imbalanced data, which is the case for the detection of relatively sparse relapses. In our data set, only 17.39% (195/1121) of events represented relapses for depression and 9.9% (63/635) represented relapses for mania. Owing to this class imbalance in the data set, it would be possible to obtain comparable accuracy values by classifying all the events as remission events (approximately 0.826 for depression and 0.900 for mania). However, this is only possible with a sensitivity of 0% and specificity of 100%. The relatively high values for sensitivity, together with the high AUROC values for both depression and mania relapse, indicate that the model correctly detects relapses.

For practical and demonstration reasons, we decided to alter the threshold probability for the model estimates to maintain 90% specificity, leading to one false alarm in 10 events. This threshold can be set to any arbitrary value based on the trade-off between false alarms and overlooked relapses.

Another consequence of the relatively rare occurrence of relapses in patients with BD is that very long observation periods are necessary to capture both the symptomatic and nonsymptomatic periods in outpatients with BD. Long-term studies investigating the relapse detection capability of self-assessment and other digital tools are therefore relatively scarce. In a cross-sectional setting (a single assessment per subject), Adler et al [20] achieved results comparable with this study: the depression or hypomania detection ability of the Affective Self-Rating Scale against a threshold reference of combined MADRS-based depression and Hypomania Interview Guide-Clinical–based hypomania showed a sensitivity of 0.90 and specificity of 0.71. It has also been shown that identification of a depressive subgroup among nonclinical samples of older adults was possible by a machine learning model using a combination of ecological momentary assessment records and actigraphy features at very high accuracy (AUROC 0.96) [11]. A similar model based on a combination of sleep and activity-related features was able to predict mood episodes in a short window of 3 days with high accuracy (AUROC between 0.79 and 0.93 in BD) [10].

Regarding comparable research in other severe psychiatric conditions, previous studies on patients with schizophrenia have identified questions targeted at basic psychosis symptoms as an efficient strategy to identify emerging relapses [44]. This strategy has also been successfully implemented by our group for schizophrenia in the Information Technology Aided Relapse Prevention Programme in Schizophrenia program [45,46]. Targeted questionnaires may thus complement passive sensing approaches and digital phenotyping in relapse detection in the future [47] and aid targeted care for patients in home environments.

In this study, the relapse prediction model was based only on the current and past week’s mood assessments. To this end, we chose a conservative approach to track the relapse risk. Alternatively, even more sophisticated models could be used to enhance relapse prediction, for example, through data transformations and advanced statistical techniques, including estimating spectral density, periodicity [48], or using various nonlinear models [49].

The data could also be combined with general relapse risk predictors, including medication, bipolarity index score, and patient demographics. As these and similar factors have been shown to predict the risk of future relapse, it is likely that their inclusion in the model should improve its performance. These approaches hold promise for future improvement of the existing models and the design of new and more efficient relapse prevention algorithms in BD.

The results presented earlier demonstrate that the relapse detection capability of ASERT is promisingly high. However, to obtain an estimate of the detection performance in a clinical setting, an additional prospective study is necessary.

### Limitations

**Figure 3:**
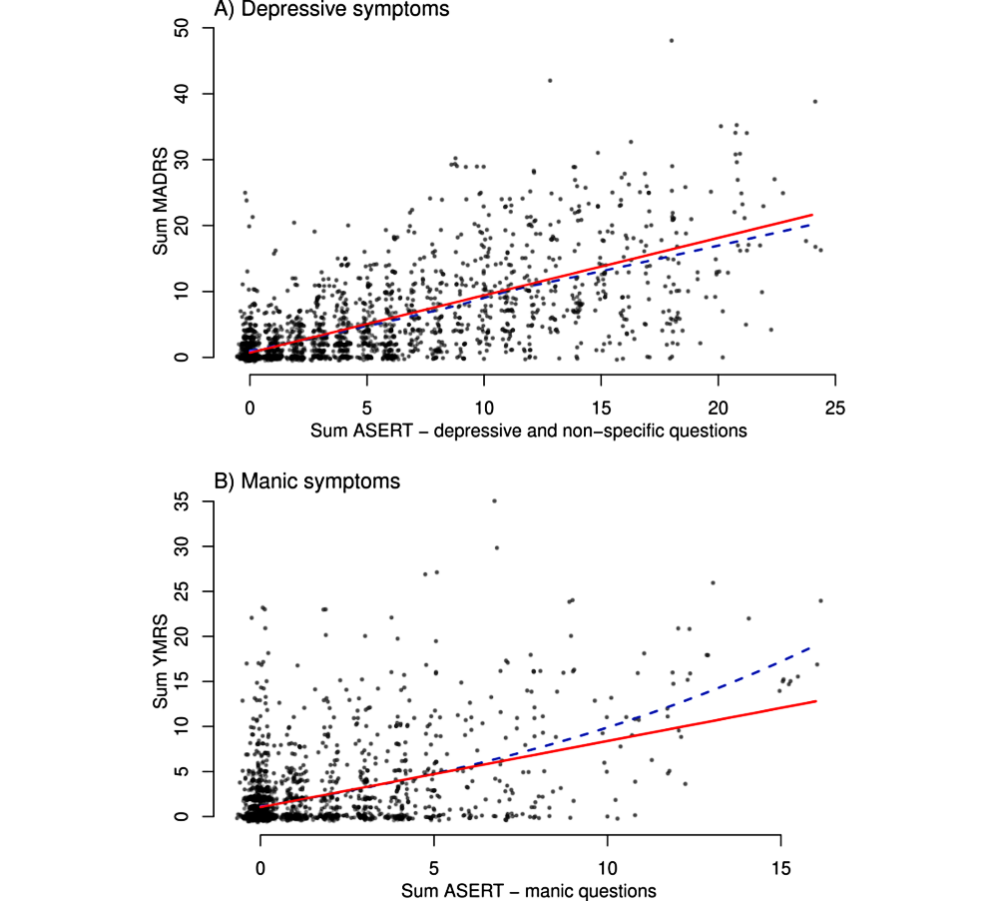
Correlation between the clinical scales and the corresponding ASERT questionnaire subscores for (A) the sum of questions about depression and nonspecific questions subscore compared with the sum of MADRS scores and (B) the questions about mania subscore compared with the sum of YMRS scores. For this visualization only, the plotted values include random jitter in both the ASERT questionnaire and MADRS or YMRS, and the points are made partially transparent. The solid red line represents the fixed-effects part of the relevant mixed-effects model, and the blue dashed line represents a smooth trend (locally estimated scatterplot smoothing). ASERT: Aktibipo Self-rating; MADRS: Montgomery-Åsberg Depression Rating Scale; YMRS: Young Mania Rating Scale.

Our study has several limitations. First, ASERT is designed to monitor subtle mood changes during remission periods and to react quickly to any worsening. Therefore, the range may not be sufficient to distinguish between different levels of more severe symptomatic periods. However, the results do not suggest saturation for the combination of depressive and nonspecific questions and show moderate saturation for mania (dashed line in Figure 3). The saturation in the ASERT questionnaire for mania manifests as nonlinearity of the relationship to the clinical scales and may have introduced a negative bias into the validation models presented earlier. The saturation thus renders our modeling results conservatively, which promises further possible improvement by nonlinear models.

Second, the MADRS and YMRS interviews with the psychologist may have affected the patient’s reflection on their state, potentially improving ASERT responses. This is not a problem when the ASERT questionnaires preceded the interview at the clinical scale. However, our analysis included both ASERT self-reports that preceded the interview and followed the clinical interview. To assess the effect of the sequence (ASERT before and after the scaling interview), we performed separate post hoc sensitivity analyses for these two cases, and the results did not differ substantially from the main analysis on all the data (Tables S3 and S4 in Multimedia Appendix 1).

Third, the definition of relapse was based only on the threshold of the total score of the MADRS or YMRS [7] and other events and factors indicating a relapse (hospitalizations, functional disability, aggression, or suicidal ideations) were not considered. The reason for this is that this information was collected retrospectively from the psychiatrists of the patients in the study, and that this information was not reliably complete for all patients at the time of the analysis, as the AKTIBIPO400 study is still ongoing. This omission likely introduces a negative bias, reducing the specificity (ie, increasing the false alarm rate) by not marking some of the weeks with low values of the scales as a relapse. The effect on sensitivity cannot be easily deduced. As the scaling is relatively frequent and regular, and the hospitalizations and other events are in good agreement with the partial data set that we already have, we consider that this issue should not represent a major source of bias for the results.

Fourth, an important issue is linked to the validation procedure: by design, data from the same patients are present in the training and testing data sets. This situation is due to the necessity to estimate the random effects of the mixed-effects model using each patient’s data, as well as the need for both remission and relapse data in both data sets for a proper evaluation. Although the MADRS and YMRS data are about a month apart, this fact may represent a positive bias to the results, and the only solution is to validate the relapse detection capabilities of the model on an independent prospective data set. In real-world deployment, the proposed approach corresponds to training a model that includes pilot data from each patient (eg, the first several months of follow-up) and evaluating the model on future data.

Fifth, the course of BD may consist of long (depressive) episodes. In such cases, many consecutive self-reports and clinical scales correspond to one event. These repeated measurements may have inflated the performance measures in relapse detection. From the point of view of statistical modeling, it is important to examine whether the models exploit the smoothness of the data relying only on the data history and self-similarity or whether the model properly treats the information in the data. To explore these properties of the data in relation to the relapse detection problem, we trained the relapse detection models on a subset of new relapses (relapses preceded by nonrelapse clinical scales). The results shown in Table S5 of Multimedia Appendix 1 indicate that the relapse detection models are not dependent on the data self-similarity and are able to detect new relapses with comparable performance to the detection of all relapses.

Finally, although the longitudinal study design, frequent self-report, and clinical assessments provided a rich data set for the analyses, the relapse detection models were based on a relatively small subset of patients with depressive or manic relapse.

### Remote Mood Monitoring

Despite the rapid trend in mobile and digital health worldwide, concerns have been raised about the effect of smartphone-based monitoring systems on the risk of relapse of affective episodes. An exploratory analysis within the MONARCA I randomized clinical trial of patients with BD suggested that smartphone-based monitoring may reduce the risk of relapse of mania but that it can possibly increase the risk of a relapse of depression [50]. However, a recent study by Faurholt-Jepsen et al [51] on the effect of a smartphone-based monitoring system on illness activity in BD (MONARCA II) showed no significant difference between the intervention and control groups in terms of the risk of relapse. In contrast, there were differences in perceived stress and quality of life in favor of the intervention group. Some efficacy studies are yet to be evaluated [16,52]. Although a validation in a randomized controlled trial (RCT) provides a high level of evidence, few mobile solutions have been validated and the overall methodological quality of the RCTs in this field is rather low [53]. Moreover, existing RCTs of monitoring systems for BD with a clinical feedback loop (ie, providing the automatic system’s output to the patient or physician) have shown mixed or unfavorable results [50,51].

There is a growing concern worldwide about privacy and user rights in the health informatics domain [54], which poses a challenge for legislators adapting to fast-developing technologies. This also poses a challenge for the patients themselves, understanding their own rights and data security policies, as well as understanding which mobile solutions in a myriad of options are of high quality and clinically validated [55]. Despite these relevant challenges in the regulatory, implementation, and educational domains, mobile apps represent a great opportunity to improve clinical practice by providing timely and widely available digital solutions.

These findings suggest that although the use of smartphone-based patient monitoring for direct decision making about further treatment may be a long and difficult journey, the feedback provided to the patients, and also to their psychiatrist, may improve the patient’s experience and quality of life. Therefore, smartphone-based monitoring systems in patients with BD could provide support for both patients and health care providers. Continuous monitoring of the patient’s condition, together with support provided by the clinician with their detailed insight into the patient’s condition could lead to increased treatment adherence in patients with BD [51] and a fine-grained and measurement-based adaptation of treatment choices by treating clinicians [19].

### Conclusions

This study presented a novel questionnaire for remote mood monitoring of patients with BD via ASERT through a mobile app. The internal structure and external validity of the ASERT were verified, and the ASERT-based model achieved high accuracy in relapse detection when used as an input into a relapse detection system. Therefore, the questionnaire provides a strong tool for remote mood monitoring in BD, calling for the preparation of a well-designed feedback system that would further extend its utility in clinical care. The simplicity, brevity, scalability, and clinical validity of ASERT make it an appropriate questionnaire for use in observational studies and, after further evaluation and validation, potentially also in routine clinical practice.

## Appendix

Multimedia Appendix 1 Supplementary information to the main text, including supplementary tables for the model selection procedure, the full table of medications used by the patients during the AKTIBIPO400 study, and results of post hoc analyses of the effect of the order of clinical and self-assessment on the main results.

## Abbreviations

ASERT: Aktibipo Self-rating
AUROC: area under the receiver operating characteristic curve
BD: bipolar disorder
LME: linear mixed-effects
MADRS: Montgomery-Åsberg Depression Rating Scale
NIMH: National Institute of Mental Health
RCT: randomized controlled trial
YMRS: Young Mania Rating Scale
