# Supplementary material for: Validity of the Aktibipo Self-rating Questionnaire for the Digital Self-assessment of Mood and Relapse Detection in Patients With Bipolar Disorder: Instrument Validation Study
Source: JMIR Ment Health. 2021 Aug 9;8(8):e26348. doi: 10.2196/26348 (PMC8386400; doi:10.2196/26348)
Supplement: Multimedia Appendix 1 [file mental_v8i8e26348_app1.docx]

# SUPPLEMENTARY INFORMATION: Validity of the Aktibipo Self-rating Questionnaire for the Digital Self-assessment of Mood and Relapse Detection in Patients With Bipolar Disorder: Instrument Validation Study

## Relapse detection: model selection and validation

The following table represents the sequence of models of increasing complexity as compared during the model selection process.

**Table S1:** General formula of the models in the model selection sequence

| 1. | $logit CSR= \beta_{SR del0}\cdot{SR}_{del0}+ \beta_{0}$ |
| --- | --- |
| 2. | $logit CSR= \beta_{SR del0}\cdot{SR}_{del0}+ \beta_{0}+b_{0\vert patient}$ |
| 3. | $logit CSR= \beta_{SR del0}\cdot{SR}_{del0}+ \beta_{0}+b_{SR del0\vert patient}\cdot{SR}_{del0}+b_{0\vert patient}$ |
| 4. | $logit CSR= \beta_{SR del0}\cdot{SR}_{del0}+\beta_{SR del7}\cdot{SR}_{del7}+ \beta_{0}+b_{0\vert patient}$ |
| 5. | $logit CSR= \beta_{SR del0}\cdot{SR}_{del0}+\beta_{SR del7}\cdot{SR}_{del7}+\beta_{SR del14}\cdot{SR}_{del14}+ \beta_{0}+b_{0\vert patient}$ |

Legend: The *CSR* indicates the clinical scale relapse logical variable - either the sum of MADRS ≥15 (outcome of the depression relapse model), or the sum of YMRS≥15 (outcome of the mania relapse model). The *SR* indicates the self-report subscore, i.e., the sum of questions about depression and non-specific questions in case of depression relapse, and the sum of questions about mania in case of mania relapse. The *β* are the coefficients in the fixed effect part of the model, *β*_0_ is the intercept. The *b* is the coefficient in the random effects part of the model, the *b_0_* is the random intercept. The subscripts *del0, del7* and *del14* indicate the self-report from the concurrent week, seven days before the scale assessment and 14 days before the scale assessment.

**Table S2:** Full table of medications used by the patients in the AKTIBIPO400 study.

|  | **All patients** | **Depression subset** | **Mania subset** |
| --- | --- | --- | --- |
|  | N = 99 | N = 51 | N = 27 |
| **Antidepressant** n (%) | 43 (43.4) | 30 (58.8) | 13 (48.1) |
| sertraline | 11 (11.1) | 8 (15.7) | 3 (11.1) |
| escitalopram | 11 (11.1) | 7 (13.7) | 3 (11.1) |
| desvenlafaxine | 8 (8.1) | 7 (13.7) | 2 (7.4) |
| trazodone | 8 (8.1) | 5 (9.8) | 2 (7.4) |
| vortioxetine | 5 (5.1) | 4 (7.8) | 1 (3.7) |
| citalopram | 4 (4.0) | 3 (5.9) | 1 (3.7) |
| mirtazapine | 4 (4.0) | 3 (5.9) | 1 (3.7) |
| paroxetine | 4 (4.0) | 2 (3.9) | 1 (3.7) |
| bupropion | 3 (3.0) | 2 (3.9) | 1 (3.7) |
| agomelatine | 3 (3.0) | 2 (3.9) | 1 (3.7) |
| amitriptyline | 2 (2.0) | 2 (3.9) |  |
| venlafaxine | 2 (2.0) | 2 (3.9) |  |
| fluoxetine | 2 (2.0) | 1 (2.0) |  |
| duloxetine | 1 (1.0) | 1 (2.0) |  |
| imipramine | 1 (1.0) | 1 (2.0) |  |
| selegiline | 1 (1.0) |  |  |
| nortriptyline | 1 (1.0) |  |  |
| clomipramine | 1 (1.0) |  |  |
| **Antipsychotic** n (%) | 76 (76.8) | 38 (74.5) | 20 (74.1) |
| quetiapine | 39 (39.4) | 19 (37.3) | 12 (44.4) |
| aripiprazole | 33 (33.3) | 19 (37.3) | 11 (40.7) |
| olanzapine | 26 (26.3) | 16 (31.4) | 8 (29.6) |
| risperidone | 5 (5.1) | 4 (7.8) | 2 (7.4) |
| haloperidol | 4 (4.0) | 3 (5.9) | 2 (7.4) |
| ziprasidone | 3 (3.0) | 2 (3.9) | 1 (3.7) |
| levomepromazine | 3 (3.0) | 1 (2.0) | 1 (3.7) |
| palipridone | 3 (3.0) | 1 (2.0) | 1 (3.7) |
| amisulpride | 2 (2.0) | 1 (2.0) | 1 (3.7) |
| cariprazine | 2 (2.0) | 1 (2.0) |  |
| clozapine | 2 (2.0) | 1 (2.0) |  |
| molindone | 1 (1.0) |  |  |
| **Anxiolytic**  n (%) | 20 (20.2) | 14 (27.5) | 7 (25.9) |
| clonazepam | 13 (13.1) | 9 (17.6) | 6 (22.2) |
| alprazolam | 6 (6.1) | 5 (9.8) | 1 (3.7) |
| bromazepam | 3 (3.0) | 2 (3.9) | 1 (3.7) |
| **Mood stabilizer and anticonvulsant** n (%) | 81 (81.8) | 46 (90.2) | 23 (85.2) |
| valproate | 32 (32.3) | 20 (39.2) | 11 (40.7) |
| lithium | 31 (31.3) | 18 (35.3) | 10 (37.0) |
| lamotrigine | 30 (30.3) | 16 (31.4) | 7 (25.9) |
| pregabalin | 5 (5.1) | 3 (5.9) |  |
| carbamazepine | 1 (1.0) | 1 (2.0) |  |
| gabapentin | 1 (1.0) |  |  |
| Eslicarbazepine acetate | 1 (1.0) |  |  |
| primidone | 1 (1.0) |  |  |
| **Hypnotic** n (%) | 5 (5.1) | 4 (7.8) | 2 (7.4) |
| zolpidem | 3 (3.0) | 3 (5.9) | 1 (3.7) |
| oxazepam | 1 (1.0) | 1 (2.0) | 1 (3.7) |
| melatonine | 1 (1.0) | 1 (2.0) |  |
| tasimelteon | 1 (1.0) |  |  |
| **Nootropic** n (%) | 2 (2.0) | 0 (0.0) | 0 (0.0) |
| methylphenidate | 2 (2.0) | 0 (0.0) |  |
| piracetam | 1 (1.0) |  |  |
| donepezil | 1 (1.0) |  |  |
| **Anticholinergic** n (%) | 1 (1.0) | 0 (0.0) | 0 (0.0) |
| biperiden | 1 (1.0) |  |  |
| **Other** n (%) | 3 (3.0) | 0 (0.0) | 0 (0.0) |
| hydroxyzine | 2 (2.0) |  |  |
| dapoxetine | 1 (1.0) |  |  |
| xanthine | 1 (1.0) |  |  |
| ergot alkaloids | 1 (1.0) |  |  |

**Legend:** The medications are divided according to their therapeutic use. The table cells contain the proportions of the patients which used the medication at any time during the study from the patient group expressed as a percent. The number in the brackets is the actual number of patients that used the medication.

## Convergent validity of the ASERT depression and mania items with MADRS and YMRS in different data subsets

In the main analysis, the convergent validity was assessed on the data set of matched clinical scales and ASERT questionnaires, which may differ by three days in either direction. However, the interview with the person performing the clinical scales may help the patient to reflect on his/her current state, and if the ASERT is submitted after the interview the patient may report a more precise assessment of his/her current state. The ASERT questionnaires submitted after the scaling may reflect not only the patient’s ability to assess his/her current state, but also an insight obtained during the interview with the scaling person. This process may introduce a bias in the statistical analyses. Therefore, we decided to examine the effect of the scaling interview on the patients’ ability to assess the mood state through the ASERT questionnaire. We performed mixed-effects model analyses of the relationship between the depressive, manic and non-specific ASERT subscores on subsets of data where the ASERT questionnaire was submitted 1) before the scaling interview, and 2) after the scaling interview. The data where the ASERT and the scaling interviews were performed on the same day were not included in any of these sets. The results are presented in Tables S3 and S4 alongside the results from the model estimated on the complete dataset. From the results on the data subsets, it is clear that the even though the coefficient estimates vary slightly, the scaling interview did not substantially affect the results. Since the subsets are considerably smaller than the complete data set (complete data set - 2159 observations, ASERT before scaling - 963 observations, ASERT after scaling - 591), the difference in the values may be attributed to higher variance of estimates on smaller data sets. However, the relationships on the subsets had the same direction and were very similar to the results on the complete data set.

**Table S3:** Results of the mixed-effects model of the relationship between the ASERT depressive and non-specific subscore to the MADRS on the complete data set and two subsets: the ASERT performed before the scaling interview subset and the ASERT performed after the scaling interview subset

| MADRS ~ ASERT_DEP NSP_ + (ASERT_DEP NSP_\| ID_PATIENT_) | | | |
| --- | --- | --- | --- |
| ALL DATA | | | |
|  |  | Coefficient | P-value |
|  | Intercept | 0.71 | 0.0002 |
|  | ASERT_DEP NSP_ | 0.87 | 0.0002 |
|  | Number of patients: | | 99 |
|  | Number of observations: | | 2159 |
| SUBSET – ASERT before scaling interview | | | |
|  |  | Coefficient | P-value |
|  | Intercept | 0.80 | 0.0002 |
|  | ASERT_DEP NSP_ | 0.87 | 0.0002 |
|  | Number of patients: | | 96 |
|  | Number of observations: | | 963 |
| SUBSET – ASERT after scaling interview | | | |
|  |  | Coefficient | P-value |
|  | Intercept | 0.96 | 0.0002 |
|  | ASERT_DEP NSP_ | 0.75 | 0.0002 |
|  | Number of patients: | | 91 |
|  | Number of observations: | | 591 |

**Legend:** DEP: depression; MADRS: Montgomery-Åsberg Depression Rating Scale; NSP: non-specific question;(ASERT_DEP NSP_| ID_PATIENT_): random intercepts and slopes

**Table S4:** Results of the mixed-effects model of the relationship between the ASERT manic subscore to the YMRS on the complete data set and two subsets: the ASERT performed before the scaling interview subset and the ASERT performed after the scaling interview subset

| YMRS ~ ASERT_MAN_ + (ASERT_MAN_ \| ID_PATIENT_) | | | |
| --- | --- | --- | --- |
| ALL DATA | | | |
|  |  | Coefficient | P-value |
|  | Intercept | 1.05 | 0.0001 |
|  | ASERT_MAN_ | 0.73 | 0.0001 |
|  | Number of patients: | | 99 |
|  | Number of observations: | | 2159 |
| SUBSET – ASERT before scaling interview | | | |
|  |  | Coefficient | P-value |
|  | Intercept | 1.11 | 0.0001 |
|  | ASERT_MAN_ | 0.96 | 0.0001 |
|  | Number of patients: | | 96 |
|  | Number of observations: | | 963 |
| SUBSET – ASERT after scaling interview | | | |
|  |  | Coefficient | P-value |
|  | Intercept | 1.34 | 0.0503 |
|  | ASERT_MAN_ | 0.54 | 0.0007 |
|  | Number of patients: | | 91 |
|  | Number of observations: | | 591 |

**Legend:** MAN: mania; (ASERT_MAN_| ID_PATIENT_): random intercepts and slopes; YMRS: Young Mania Rating Scale

## Abilities of the relapse detection models to detect only new relapses

The course of BD may consist of long episodes, especially in the case of depression. Long episodes of depressive mood may inflate the relapse detection model performance. A depressive episode lasting several months is sampled with multiple clinical scales and self-report questionnaires, however the actual values may be very similar. The detection of a change from a remission state into a relapse is very different from detecting all events which would classify as a relapse according to MADRS or YMRS clinical scale. The benefit of detecting new relapses is especially crucial in the frequent continuous monitoring of mood in BD. The detection of the deterioration of patient’s state is a key moment for the rapid response from the clinician or other caregivers to provide the appropriate treatment for the patient.

Another question is whether the ASERT questionnaire substantially improves the relapse detection in comparison to the information already known from the previous MADRS/YMRS scale. Even though this scenario may not be relevant to the use of ASERT questionnaire in the real world, where the clinical scales will not be generally collected, it is important to evaluate this effect for better understanding of how the relapse detection with the ASERT corresponds to short-term and long-term changes in mood.

In order to examine the abilities of the proposed relapse detection system to detect the new relapses we created a subset of the original dataset where we kept the events classified as relapses only if the preceding event was classified as remission (MADRS < 15 or YMRS < 15) , all the events denoted as remissions were kept. In the case of the new depressive relapses, the subset consisted of 1081 observations, 1012 remissions and 69 new relapses, while in the case of the new manic relapse, the subset consisted of 994 observations, 953 remissions and 41 new relapses. We trained models for depressive and manic relapse detection on the new relapses subsets. Because the response variables are different from those used in the main analysis it is not possible to compare the resulting performance measures in the pairwise manner as in the case of the model selection. The resulting performance measures of the modes on new relapses subsets are reported in the Table S2.

**Table S5:** Performance of the ASERT in detecting new depressive and new manic relapse

| DEPRESSIVE RELAPSE DETECTION | $MADRS\geq15 \sim{ASERT}_{DEPNSP, current week}+{ASERT}_{DEPNSP, previous week} + (1 \vert{ID}_{patient})$ | | | | |
| --- | --- | --- | --- | --- | --- |
|  | Set | Accuracy | AUC | Sensitivity | Specificity |
|  | training | 0.892 | 0.927 | 0.756 | 0.902 |
|  | testing | 0.870 | 0.843 | 0.560 | 0.892 |
| MANIC RELAPSE DETECTION | $YMRS\geq15 \sim{ASERT}_{MAN, current week} + (1\vert{ID}_{patient})$ | | | | |
|  | Set | Accuracy | AUC | Sensitivity | Specificity |
|  | training | 0.901 | 0.957 | 0.852 | 0.902 |
|  | testing | 0.897 | 0.890 | 0.75 | 0.900 |

Legend:

AUC: area under ROC curve; DEP: depression; MADRS: Montgomery-Åsberg Depression Rating Scale; MAN: mania; NSP: non-specific question; $(1 | {ID}_{patient})$: random intercept; YMRS: Young Mania Rating Scale

*Depressive relapse*: The results of the best scoring logistic mixed-effects model in the detection of a depression relapse from the sum of the depression-related questions and the non-specific questions from the current week and from the previous week. *Manic relapse*: Results of the best scoring logistic mixed-effects model in the detection of a mania relapse used the sum of the mania-related questions from the current week.

Note that the detection thresholds for all models were adjusted to specificity 90%, using the ROC curve of the training set.

In comparison to the relapse detection results in the main analysis (see Table 5), the results when detecting new relapses do not substantially deviate. The detection of new depressive relapses is slightly worse than the detection of all relapses (new relapses AUC=0.843, all relapses AUC=0.880), while in the case of the new manic relapses the detection is slightly better (new relapses AUC=0.890, all relapses AUC=0.844). With the subset of new relapses we eliminated the effect of long episodes of mood. The results indicate that the models are detecting the relapses better than just copying the patient’s history (eg guessing the current relapse from previous scale) or the patient’s propensity to severe mood states (eg modelling only the prior probability of relapse).
